# Genetic Determinants Associated With the Biofilm Formation Impairment in *Pseudomonas aeruginosa* Clinical Isolates

**DOI:** 10.1002/mbo3.70168

**Published:** 2025-12-10

**Authors:** Andrei V. Vvedenskii, Alina S. Ivkina, Dmitry N. Konanov, Tatiana A. Savinova, Ludmila S. Fedorova, Elena N. Ilina

**Affiliations:** ^1^ Department of Molecular Biology and Bioinformatics Institute for Systems Biology and Medicine of Rospotrebnadzor Moscow Russia

**Keywords:** biofilms, DNA sequencing, genome analysis, *Pseudomonas aeruginosa*

## Abstract

*Pseudomonas aeruginosa* is a model organism for biofilm formation research, as it forms biofilms under diverse environmental conditions. At the same time, numerous studies have reported impaired‐biofilm formation in clinical isolates; however, the genetic basis of these impairments remains unexplored. In this study, we assessed the ability of *P. aeruginosa* clinical isolates from a laboratory collection to form biofilms. Among these isolates, three demonstrated biofilm formation impairment. A comparative genomic analysis revealed genetic determinants associated with biofilm formation impairment, including mutations in the *pelA* and *fleQ* genes, and *psl* operon deletion. Interestingly, the identified loss‐of‐function mutations in regulatory genes involved in biofilm formation did not appear to affect the ability to form biofilms.

## Graphical abstract

Numerous studies have reported biofilm formation impairment in *Pseudomonas aeruginosa* clinical isolates, however, the genetic basis of this phenomenon remains largely unexplored. In this study, we aimed to identify associations between impaired‐biofilm formation and loss‐of‐function mutations in biofilm regulatory and effector genes.

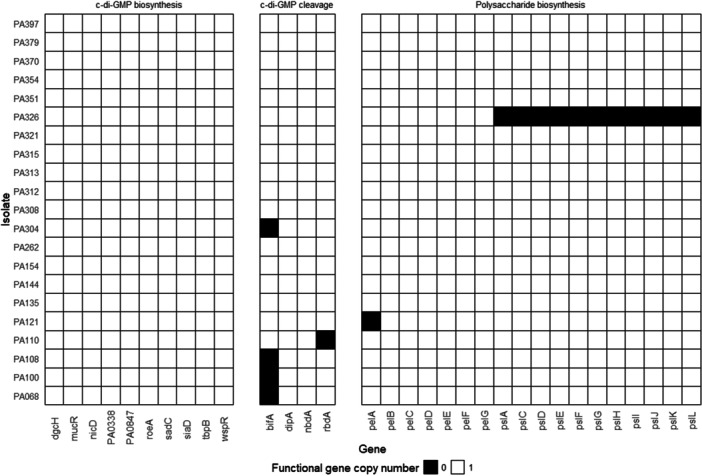

## 1 Introduction

*Pseudomonas aeruginosa* is one of the major nosocomial pathogens that colonizes diverse anatomical sites with particularly high prevalence in cystic fibrosis patients, burn wound cases, and immunocompromised individuals (Lazareva et al. 2015; Bodey et al. 1983; Qin et al. 2022). Infections caused by this pathogen often follow a chronic course and are notoriously difficult to treat due to its intrinsic antibiotic resistance (Lazareva et al. 2015; Bodey et al. 1983; Qin et al. 2022). A key factor complicating treatment is *P. aeruginosa*'s ability to form biofilms (Maurice et al. 2018; Qin et al. 2022; Tuon et al. 2022). Bacterial cells within biofilms acquire resistance to antibiotics (Ciofu and Tolker‐Nielsen 2019; Thöming and Häussler 2022) and disinfectants (Fedorova and Ilyakova 2023; Mah and O'Toole 2001).

*P. aeruginosa* transitions from planktonic to sessile mode inside the self‐produced extracellular matrix during biofilm formation (Sauer et al. 2002). In the first stage of this process, the planktonic bacterial cells attach and start to move across the surface by the type IV pili and produce the biofilm matrix (O'Toole and Kolter 1998). In the later stages, cells form conglomerates or microcolonies, embedded in the intercellular matrix, forming a mature biofilm (Sauer et al. 2002).

The main organic components of the biofilm matrix formed by *P. aeruginosa* are polysaccharides, proteins, and extracellular DNA, with polysaccharides as a major part of the matrix (Flemming et al. 2021; Wickramasinghe et al. 2020; Wingender et al. 2001). While alginate is an important virulence factor in chronic lung infections (Ramsey and Wozniak 2005), it is not essential for biofilm formation (Wozniak et al. 2003). In contrast, at least one of the Pel or Psl polysaccharides is required for biofilm development (Harmsen et al. 2010).

The key intracellular regulator of biofilm formation is cyclic dimeric guanosine monophosphate (c‐di‐GMP) (Park and Sauer 2022; Valentini and Filloux 2016). Genes responsible for biofilm formation can be divided into two functional groups: regulatory genes, which modulate intracellular c‐di‐GMP level in response to environmental cues, and effector genes, which control biofilm matrix synthesis and cell motility.

Regulatory genes primarily include genes of diguanylate cyclases and phosphodiesterases, which control the level of c‐di‐GMP. Diguanylate cyclases with a single catalytic GGDEF domain synthesize c‐di‐GMP in response to external stimuli, while phosphodiesterases with two catalytic domains, GGDEF and EAL, where the GGDEF domain usually has a regulatory role and the EAL domain is responsible for catalytic activity, cleave c‐di‐GMP (Dahlstrom and O'Toole 2017; Park and Sauer 2022; Valentini and Filloux 2016). It has been experimentally proven that the overexpression of diguanylate cyclase genes *siaD*, PA0338, *tbpB* (*yfiN*), *wspR*, *nicD*, *sadC*, PA0847, *roeA*, and *dgcH* (Bhasme et al. 2020; Kulesekara et al. 2006), as well as the knockout of phosphodiesterase genes *dipA*, *bifA*, *rbdA*, *nbdA* enhances biofilm formation (Cai et al. 2020; Ha et al. 2014; Kuchma et al. 2007; Kulesekara et al. 2006; Roy et al. 2012). The *mucR* gene has two catalytic domains, which is more common for phosphodiesterases, but exhibits diguanylate cyclase activity and enhances biofilm formation in vitro (Kulesekara et al. 2006).

As the main polysaccharides in biofilm are Pel and Psl (Colvin et al. 2012), we focused on the genes responsible for the synthesis of these polysaccharides as the main effector genes of biofilm development. Pel and Psl polysaccharide synthesis genes are organized in two operons. The *pel* operon consists of seven genes: *pelA*, *pelB*, *pelC*, *pelD*, *pelE*, *pelF*, *pelG* (Mann and Wozniak 2012) and the *psl* operon consists of 15 genes: *pslA*, *pslB*, *pslC*, *pslD*, *pslE*, *pslF*, *pslG*, *pslH*, *pslI*, *pslJ*, *pslK*, *pslL*, *pslM*, *pslN*, *pslO*, 11 of them are essential for Psl biosynthesis, while four Psl genes, *pslB*, *pslM*, *pslN*, and *pslO*, are not required (Byrd et al. 2009).

An increase in the c‐di‐GMP level activates the promoters of *pel* and *psl* operones, thereby initiating the synthesis of matrix polysaccharides (Park and Sauer 2022). At the low level of c‐di‐GMP, *pel* and *psl* promoters are inactivated by the transcriptional regulator FleQ (Baraquet and Harwood 2016). c‐di‐GMP binds to the FleQ‐FleN protein complex at increased concentrations, leading to a conformational change in the complex, which switches its activity from a transcriptional repressor to a transcriptional activator (Baraquet and Harwood 2016; Torres‐Sánchez et al. 2023).

To estimate the quantity of biofilm formed, the microtiter dish biofilm formation assay is usually applied (O'Toole 2011). Biofilms are grown during 8–24 h incubation and stained by crystal violet (CV) solution with subsequent extraction and the quantitative measurement of the binding dye (Banerjee 2014; Elnegery et al. 2021; Ghadaksaz et al. 2015; Lima et al. 2017; Smith et al. 2006). Through different estimations from 4% (Elnegery et al. 2021) to 49% (Ghadaksaz et al. 2015) of *P. aeruginosa* clinical isolates do not form biofilm according to this technique. In addition, the level of biofilm formation decreases during lung infection in patients with cystic fibrosis (Smith et al. 2006). At the same time, the question about genetic variants responsible for disturbance in biofilm formation remains open. Ghadaksaz and coauthors showed that the *pslA* gene was deleted in 16.3% of clinical isolates and the *pelA* gene was deleted in 54.8% of isolates (Ghadaksaz et al. 2015). Additionally, *pelA* gene deletion was correlated with the absence of biofilm formation (Ghadaksaz et al. 2015). In a more recent paper, no correlation between the deletion of the *pelA* gene and biofilm formation was observed (Płókarz et al. 2022).

The purpose of our research was to correlate genetic variations to impairment of biofilm formation in *P. aeruginosa* clinical isolates obtained from Moscow clinics. We employed whole‐genome sequencing followed by comparative genomic analysis of biofilm‐forming and nonbiofilm‐forming isolates, with a particular focus on selected regulatory and effector genes involved in biofilm formation. The identified variants were cross‐referenced with the National Center for Biotechnology Information (NCBI) Genome database (Sayers et al. 2025) to assess their global prevalence.

## 2 Materials and Methods

### 2.1 Bacterial Isolates and Culture Conditions

*P. aeruginosa* isolates (*n* = 21) were obtained from clinical specimens (from sputum, urine, respiratory tract, wound, and bile) and the hospital environment in Moscow clinics between 2017 and 2023 (Table 1) and identified using matrix‐assisted laser desorption/ionization time‐of‐flight mass spectrometry (MALDI‐TOF MS). The isolates are stored as glycerol stocks (50% glycerol) in the laboratory collection at −80°C. For further analysis, isolates were transferred to hydrolysate of fish meal (GFM) broth (State Research Center for Applied Biotechnology and Microbiology, Obolensk) and incubated overnight at 37°C. After incubation, samples were plated on GFM agar and incubated again overnight at 37°C. Petri dishes were then stored at +4°C for future use.

**Table 1:** Origin of *Pseudomonas aeruginosa* isolates used in this study.

Isolation source	Number of isolates	Isolates
Catheter	4	PA144, PA308, PA312, PA315
Bile	3	PA313, PA321, PA326
Bronchial lavage	3	PA110, PA321, PA354
Sputum	3	PA108, PA121, PA397
Hospital equipment	2	PA370, PA379
Hospital surfaces	2	PA068, PA100
Unknown	2	PA154, PA262
Urine	1	PA135
Wound	1	PA304
Total	21	

### 2.2 Biofilm Assay

The microtiter plate biofilm assay was performed as described by O'Toole (2011) with modifications. Briefly, a single colony was inoculated into 3 mL of GFM broth and incubated overnight at 37°C with shaking (180 rpm). After incubation, cultures were diluted 1:100 in fresh GFM broth, and 100 µL aliquots were transferred in triplicate to a 96‐well nontreated polystyrene plate (Wuxi NEST Biotechnology Co. Ltd). The plate was incubated at 37°C (90% humidity) for 24 h. Wells were washed twice with 200 µL of distilled water to remove planktonic cells, then stained with 125 µL of 0.1% (w/v) CV for 10 min at room temperature. Excess stain was removed by three washes with distilled water. To solubilize biofilm‐associated dye, 125 µL of 30% acetic acid was added per well for 10 min. A 100 µL aliquot of the solubilized CV was transferred to a new plate, and absorbance was measured at 550 nm (OD550) using a microplate reader (FlexA‐200, Hangzhou Allsheng Instruments). The mean OD550 of triplicate wells was calculated for each isolate, with sterile GFM broth as a blank. Three biological replicates were performed. As a positive control for biofilm formation, the biofilm‐producing *P. aeruginosa* strain ATCC 15442 (Cole et al. 1989) was used.

### 2.3 DNA Purification and Sequencing

Total DNA was extracted from 3 mL of overnight cultures (GFM broth, 37°C without shaking) using the D‐cells DNA isolation kit (BiolabMix) according to the manufacturer's instructions. DNA concentration was quantified using a Qubit 3.0 fluorometer (Thermo Fisher Scientific). For shotgun metagenomic sequencing, 100 ng of purified DNA from each sample was processed with the MGIEasy Fast FS DNA Library Prep Set (MGI Tech Co. Ltd.) to construct sequencing libraries. DNA nanoballs were prepared using the DNBSEQ‐G400RS High‐throughput Sequencing Kit (MGI). Sequencing was performed on the DNBSEQ‐G400 (MGI) platform in 2 × 150 bp paired‐end mode.

### 2.4 Genome Data Analysis

Sequencing data in FASTQ format were merged from four generated files for downstream analysis. De novo genome assembly was performed using SPAdes v3.15.5 (Prjibelski et al. 2020) with default parameters. Raw reads were aligned to the PAO1 reference genome (Stover et al. 2000) using BWA‐MEM (H. Li and Durbin 2009), followed by SAMtools processing (Danecek et al. 2021) for BAM file generation. BAM files were analyzed in Integrative Genomics Viewer to map deletion boundaries (Robinson et al. 2011). Single‐nucleotide polymorphism (SNP) calling and functional annotation were performed using BCFtools (Danecek et al. 2021) and snpEff (Cingolani et al. 2012).

### 2.5 Phylogenetic Analysis

Sequence typing, marker gene extraction, and concatenation were performed using MLSTcheck (J. Page et al. 2016). Core SNPs conserved across all genomes were identified and concatenated with kSNP4 using a k‐mer length of 21 bp (Gardner and Hall 2013; Hall and Nisbet 2023). Multiple sequence alignment and phylogenetic tree construction were performed using multiple alignment using fast Fourier transform (MAFFT) with default parameters (Katoh et al. 2019). Resulting trees were visualized and annotated using ggtree (Yu et al. 2017) and ggtreeExtra (Xu et al. 2021) R packages.

### 2.6 Searching in Databases

To estimate the prevalence of the identified mutations in *P. aeruginosa*, 1043 complete genomes were downloaded from the NCBI Genome database (Sayers et al. 2025). The target genes were extracted and aligned using MAFFT (Katoh et al. 2019) with default parameters, and the positions of interest were checked to identify the same SNPs as in our strains.

The presence of the *psl* operon in external genomes was checked using basic local alignment search tool, nucleotide (BLASTn) (Camacho et al. 2009). The nucleotide sequence of the full *psl* operon was used as the query sequence. Genomes with less than 10% coverage of the query sequence length were considered to have a similar deletion.

Sequence types of external genomes were determined using the MLST tool (Jolley et al. 2018).

## 3 Results

### 3.1 Evaluation of Biofilm Formation in *Pseudomonas* Isolates

**Figure 1:**
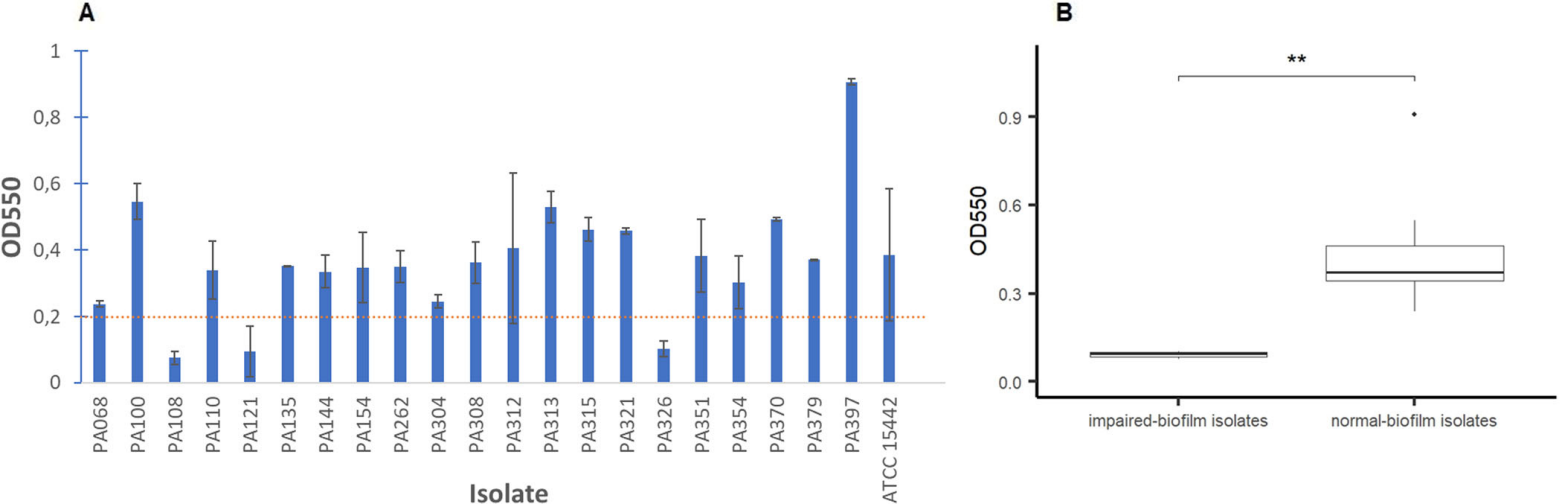
Biofilm formation levels of *Pseudomonas aeruginosa* isolates (*n* = 3 experimental replicates), measured using the CV microtiter plate assay. OD550, the average optical density of the dye extracted from biofilms at a wavelength of 550 nm. (A) Biofilm formation of individual *P. aeruginosa* isolates. The dashed line (OD550 = 0.2) indicates the threshold between normal‐biofilm and impaired‐biofilm isolates. Error bars represent the standard deviation. (B) Boxplots showing the distribution of mean OD550 values for normal‐biofilm and impaired‐biofilm isolates. Each box represents the interquartile range (IQR), the horizontal line indicates the median, and whiskers extend to 1.5 × IQR. Statistical significance between the groups was assessed using the Mann–Whitney *U* test (*p* ≤ 0.01). CV, crystal violet.

We measured the level of biofilm formation using CV staining (O'Toole 2011). Following staining, the OD550 values ranged from 0.1 to 0.9 after blank subtraction (blank OD550 = 0.1) (Figure 1A). The ATCC 15442 strain was used as a positive control, yielding an OD550 of 0.4. Three isolates, PA108, PA121, and PA326, exhibited outlier OD550 values of approximately 0.1 and were classified as impaired‐biofilm isolates. The threshold value for impaired‐biofilm isolates was set at 0.2, in agreement with previous studies (Ghadaksaz et al. 2015; Wakimoto et al. 2004). On the basis of this threshold, *P. aeruginosa* isolates were divided into two groups: normal‐biofilm producers and impaired‐biofilm producers (Figure 1B). The difference between these groups was statistically significant (*p* = 0.0013, Mann–Whitney *U* test). The remaining isolates were classified as normal‐biofilm producers, with a mean OD550 of 0.4. One isolate, PA397, formed an extremely strong biofilm, with an OD550 more than twice the average value.

To further investigate biofilm formation impairment, we questioned whether the isolates with impaired biofilm belong to the same or different phylogenetic groups and attempted to find phylogenetically close isolates with normal‐biofilm formation to impaired‐biofilm isolates.

### 3.2 Phylogenomic Analysis and Unique SNPs Determination

**Figure 2:**
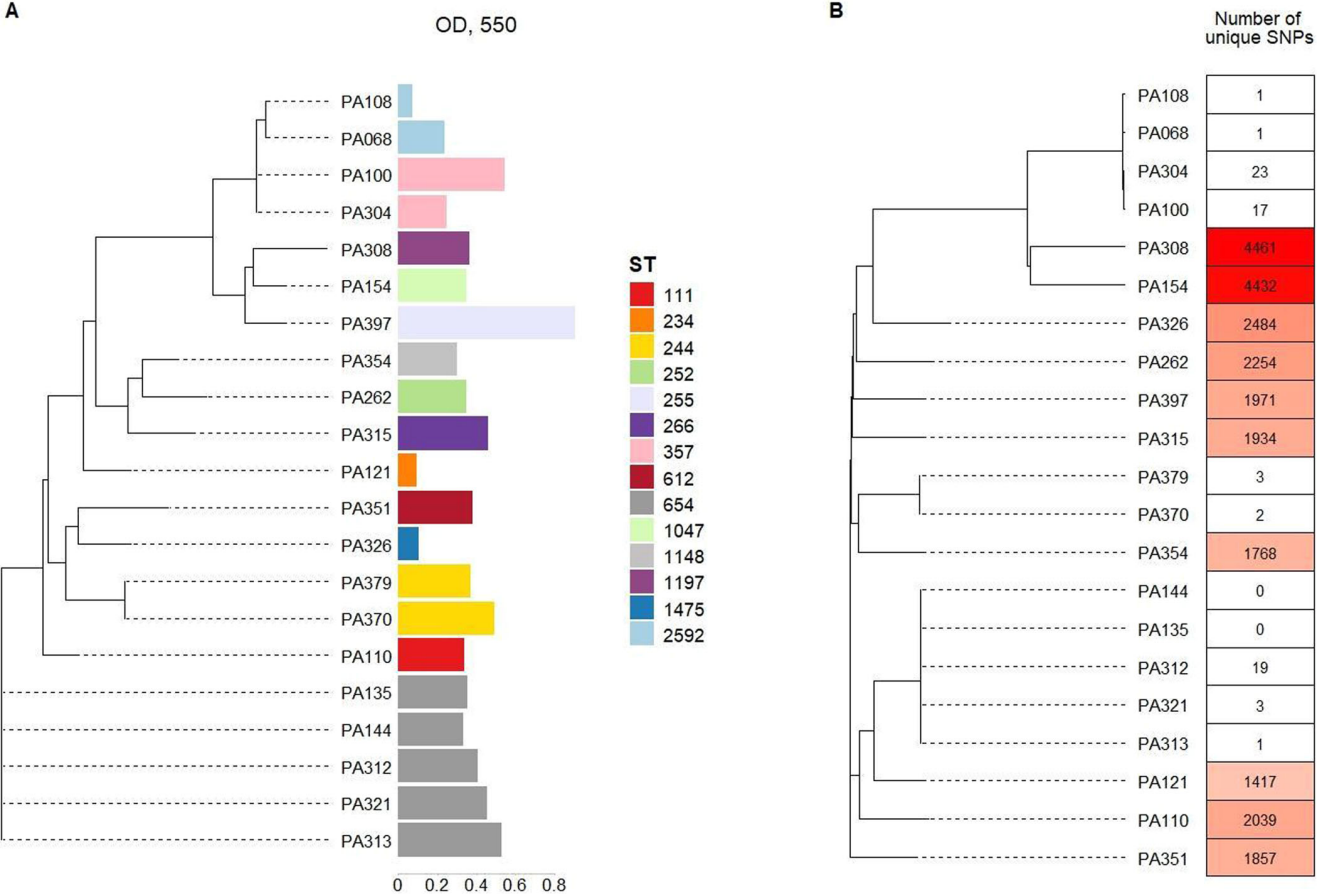
Phylogenetic trees were constructed by the neighbor‐joining method. (A) Phylogenetic tree based on the MLST concatenated sequences with added biofilm staining and ST data. (B) Phylogenetic tree based on SNPs in k‐mers with the number of unique SNPs for each isolate. MLST, multilocus sequence type; OD, optical density; SNP, single‐nucleotide polymorphism; ST, sequence type.

We produced draft genomes of 21 *P. aeruginosa* isolates from our laboratory collection. Genome assembly statistics and metadata are available in Appendix 1. We identified 15 multilocus sequence types (Curran et al. 2004; Jolley et al. 2018) (MLST or ST) of *Pseudomonas* and used concatenated sequences of ST markers to create the phylogenetic tree (Figure 2A). Three isolates with biofilm formation impairment belong to different STs: 234, 1475, and 2592. The isolate PA108 with impaired‐biofilm shares the same ST2592 as the normal‐biofilm‐producing isolate PA068. This multidrug‐resistant ST was first described in samples received from Moscow pediatric clinics (Savinova et al. 2018).

To further distinguish isolates within one sequence type, the phylogenetic analysis was performed using the kSNP4 program (Gardner and Hall 2013; Hall and Nisbet 2023). At the first stage of processing, genomes were divided into k‐mers containing SNPs in the center. The phylogenetic tree was created based on the alignment of artificial sequences obtained by combining the SNPs (Figure 2B). According to the kSNP4 results, the three *P. aeruginosa* isolates with impaired‐biofilm formation ability are located in different clades. Isolate PA326 contains 2484 unique SNPs, isolate PA121 has 1417 unique SNPs, and isolate PA108 possesses only one unique SNP and is closely related to isolate PA068, which forms a normal biofilm.

### 3.3 Identification of the Genetic Differences Between the Normal‐Biofilm‐Forming Isolate PA068 and the Impaired‐Biofilm Isolate PA108

We identified one unique SNP in the PA108 isolate using the kSNP4 program (Gardner and Hall 2013; Hall and Nisbet 2023). This SNP is located in the CAGTTCGCCG.CCCCGATCAC k‐mer. Isolate PA108 has the CAGTTCGCCG ***C*** CCCCGATCAC (with the SNP marked in italic bold font) k‐mer variant, and isolate PA068 has the CAGTTCGCCG ***A*** CCCCGATCAC k‐mer variant. A subsequent search for this k‐mer in the PAO1 genome (Stover et al. 2000) using the NCBI Nucleotide database web interface (Sayers et al. 2025) showed that it mapped to the *fleQ* gene sequence. The PA108 k‐mer variant corresponds to the c.1148T > G polymorphism in *fleQ* and resulting in a V383G substitution. To validate this finding, we conducted SNP calling with BCFtools (Danecek et al. 2021) (see Section 2). This analysis also detected only one unique SNP in the *fleQ* gene of PA108 compared with PAO1 and PA068. Thus, both methods—k‐mer‐based SNP detection and alignment‐based SNP calling—identified the c.1148T > G variant in *fleQ*.

### 3.4 Identification of Loss‐of‐Function Genetic Variants in the Genes Responsible for Biofilm Formation

To identify the genetic causes of biofilm formation impairment in the PA121 and PA326 isolates, the main effector and regulator genes of biofilm formation were screened. Only clear loss‐of‐function variants were considered, including full gene deletions, nonsense SNP mutations, and frameshift mutations.

#### 3.4.1 Identification of Loss‐of‐Function Genetic Variants in the Genes Responsible for c‐di‐GMP Turnover

**Figure 3:**
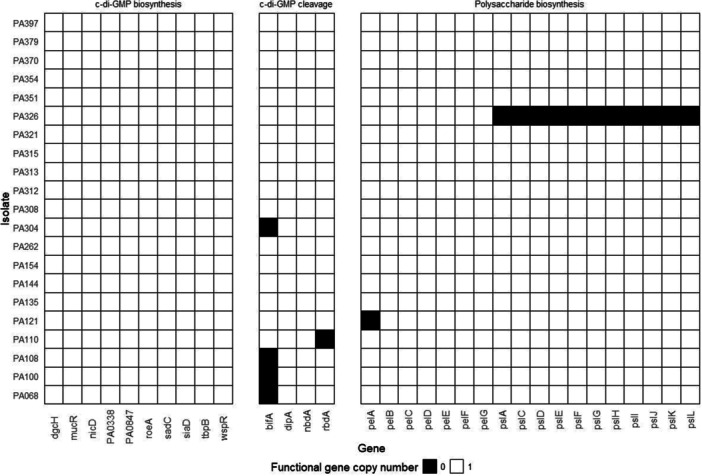
Mutations in genes involved in biofilm formation and regulation. The black squares denote full gene deletions or other loss‐of‐function mutations, such as nonsense SNP mutations and single‐nucleotide deletions. c‐di‐GMP, cyclic dimeric guanosine monophosphate; SNP, single‐nucleotide polymorphism.

Searching for loss‐of‐function mutations was performed on 10 genes encoding diguanylate cyclases (*siaD*, PA0338, *tbpB* [*yfiN*], *wspR*, *nicD*, *sadC*, PA0847, *roeA*, *dgcH, mucR*) and four genes encoding phosphodiesterases (*dipA*, *bifA*, *rbdA*, *nbdA*) with proven effects on biofilm formation (Bhasme et al. 2020; Cai et al. 2020; Ha et al. 2014; Kuchma et al. 2007; Kulesekara et al. 2006). No deletions, frameshift mutations, or nonsense mutations were found in genes encoding c‐di‐GMP synthases (Figure 3).

Frameshift mutations were identified in two of the four screened genes encoding c‐di‐GMP phosphodiesterases (Figure 3). A deletion of a G nucleotide at position 1884 in the *bifA* gene was found in four phylogenetically close isolates, PA068, PA100, PA108, and PA304 (Table 2), resulting in a frameshift of the coding sequence at G629. The resulting protein is 633 amino acids in length, compared with 699 amino acids in the reference variant. We also identified a deletion of a G nucleotide at position 792 in the *rbdA* gene in the PA110 isolate, which results in a frameshift of the translated sequence at G266 (Table 2).

**Table 2:** Loss‐of‐function variants of nucleotide sequences in genes responsible for c‐di‐GMP turnover.

Isolate	Coordinate in the genome (PAO1)	Gene	cDNA position	Amino acid substitution	PAO1#	Description of genetic variant
PA068, PA100, PA108, PA304	4,896,341 TG > T	*bifA*	c.1884delG	p.G629fs	PA4367	Frameshift variant
PA110	940,907 AG > A	*rbdA*	c.792delG	p.G266fs	PA0861	Frameshift variant

Abbreviations: c‐di‐GMP, cyclic dimeric guanosine monophosphate; cDNA, complementary DNA.

#### 3.4.2 Identification of Loss‐of‐Function Variants in the Genes Responsible for Pel and Psl Polysaccharide Biosynthesis

We performed screening for deleterious gene variants involved in Pel and Psl biosynthesis in the whole‐genome sequencing data (Figure 3). The list of genes includes 11 genes in the *psl* operon responsible for Psl biosynthesis (*pslA*, *pslC*, *pslD*, *pslE*, *pslF*, *pslG*, *pslH*, *pslI*, *pslJ*, *pslK*, *pslL*) (Byrd et al. 2009) and seven genes in the Pel biosynthesis operon (*pelA*, *pelB*, *pelC*, *pelD*, *pelE*, *pelF*, *pelG*) (Mann and Wozniak 2012).

We identified two deleterious mutations: the deletion of the entire *psl* operon in isolate PA326 and the point mutation C1609A in the *pelA* gene of the PA121 isolate, which results in the nonsense mutation E537* (Figure 3 and Table 3). These two isolates showed impaired‐biofilm formation, which is associated with the identified mutations.

**Table 3:** Loss‐of‐function variants of nucleotide sequences in genes responsible for Pel and Psl polysaccharide biosynthesis.

Isolate	Coordinate in the genome (PAO1)	Gene	cDNA position	Amino acid substitution	PAO1#	Description of genetic variant
PA326	2,439,002–2,603,339	137 genes, including the *psl* operon				Extended deletion
PA121	3,432,283 С > A	*pelA*	c.1609 C > A	p.E537*	PA3064	Nonsense mutation

Abbreviation: cDNA, complementary DNA.

### 3.5 Searching for Identified Loss‐of‐Function Variants in the NCBI Database

We searched for identified variants in the NCBI Genome database (Sayers et al. 2025). The first identified variant, the c.1148T > G SNP in the *fleQ* gene, was found in only one genome, GCF_014155905.1, with a novel β‐lactamase (X. Li et al. 2023).

The frameshift mutations in the *rbdA* and *bifA* genes were identified in 35 and 15 complete genomes, respectively (3.4% and 1.4% of analyzed genomes). While the *rbdA* frameshift mutation c.792delG is present in a broad range of sequence types, the *bifA* frameshift mutation c.1884delG is unique to ST357 and ST2592.

The deletion of the *psl* operon with the same genomic coordinates was not detected in the database. However, deletions of Psl synthesis genes and adjacent regions occur in several genomes, indicating that the *psl* operon deletion arises in clinical isolates. The well‐characterized *Pseudomonas* clinical strain PA14 harbors the partial deletion of the Psl synthesis gene cluster (Colvin et al. 2011).

The last identified variant, the c.1609 C > A SNP in the *pelA* gene, was not detected in the NCBI complete genomes.

## 4 Discussion

Numerous studies report impaired‐biofilm production in *P. aeruginosa* clinical isolates (Banerjee 2014; Elnegery et al. 2021; Ghadaksaz et al. 2015; Kunwar et al. 2021; Lima et al. 2017; Płókarz et al. 2022), with the proportion of biofilm‐producing isolates ranging from 4% (Elnegery et al. 2021) to 49% (Ghadaksaz et al. 2015). Genetic causes of biofilm formation impairment may be different. They may include mutations in effector genes involved in biofilm matrix biosynthesis as well as mutations in regulatory genes that control the expression of these effector genes. In our study, we identified three (14%) *P. aeruginosa* isolates with biofilm formation impairment (Figure 1). For all isolates, we identified the most likely genetic variants that may impair biofilm formation.

### 4.1 Identified Loss‐of‐Function Genetic Variants in the Genes Responsible for c‐di‐GMP Cleavage Have No Impact on Biofilm Formation

There are 14 genes related to c‐di‐GMP turnover that impact biofilm formation (10 guanylate cyclases and four phosphodiesterases) (Bhasme et al. 2020; Cai et al. 2020; Ha et al. 2014; Kuchma et al. 2007; Kulesekara et al. 2006). We identified two loss‐of‐function mutations in the phosphodiesterase genes, responsible for c‐di‐GMP cleavage (Table 2 and Figure 3). G266fs mutation in the *rbdA* gene of isolate PA110, located in the PAS domain upstream of the EAL domain (Liu et al. 2018), leads to translation termination 12 codons after the mutation. As a result, a truncated protein without any catalytic domain is produced from this gene variant. Inactivation of RbdA in *P. aeruginosa* PAO1 has been associated with hyper‐biofilm formation (An et al. 2010) and matrix overproduction (Cai et al. 2020), while in *P. aeruginosa* PA14, reports vary between biofilm hyperproduction (Kulesekara et al. 2006) and no effect from *rbdA* gene inactivation (Ha et al. 2014). In our case, the isolate with a functional deletion of *rbdA* showed biofilm OD550 of 0.34, which is close to the average OD550 of biofilm‐producing isolates (0.41). This suggests that in our isolate, the absence of a full *rbdA* gene does not affect biofilm formation.

Another identified variant is a frameshift mutation of the *bifA* gene (Table 2 and Figure 3). This mutation leads to the formation of a protein shortened by 66 amino acids, with truncation of the catalytic EAL domain responsible for c‐di‐GMP cleavage. The removal of part of the EAL domain involves the central β‐barrel of this domain and presumably leads to the disruption of phosphodiesterase function. The identified variant was found in four isolates, one of which demonstrated biofilm formation impairment. The average biofilm OD550 for the three biofilm‐forming isolates carrying the *bifA* frameshift mutation (excluding the fourth isolate with both *bifA* and *fleQ* mutations) was 0.34. At the same time, authors report stronger biofilm formation in the PA14 strain variant with *bifA* deletion (Kuchma et al. 2007; Kulesekara et al. 2006).

The experimental data support the idea of distinct subsystems within the global c‐di‐GMP and biofilm regulatory networks, suggesting that specific stimuli may be required to activate different proteins. Also, the activity of c‐di‐GMP–regulating enzymes may be strain‐specific (Eilers et al. 2022). The absence of the functional *rbdA* and *bifA* genes in the found clinical isolates might be compensated by the higher activity of other phosphodiesterases, or these proteins may not be active in the described isolates under the tested conditions.

### 4.2 Identified Mutations Associated With Biofilm Formation Impairment

In isolate PA121, we identified a nonsense mutation (p.E537*) in the *pelA* gene. The PelA protein consists of 948 amino acids and is divided into two domains: a glycoside hydrolase family 166 domain at the N‐terminus (amino acids 47–303) and a deacetylase domain at the C‐terminus (amino acids 520–800) (Razvi et al. 2023). The E537‐mutated protein lacks a functional deacetylase domain, which is essential for biofilm production (Colvin et al. 2013) and Pel biosynthesis, although the details of Pel polysaccharide processing by PelA remain unknown. The hydrolase domain is crucial for cleaving Pel polymer into smaller fragments, thereby generating a low molecular weight secreted form of Pel (Razvi et al. 2023). Exogenous recombinant forms of the PelA hydrolase domain have been shown to disrupt Pel‐dependent biofilms (Baker et al. 2016). To our knowledge, intracellular expression of the isolated hydrolase domain has not been previously reported. We hypothesize that the PelA variant lacking the deacetylase domain may possess increased cleavage activity, leading to reduced cell adherence and weak biofilm formation.

In the impaired‐biofilm‐forming isolate PA326, an extended 164,337 bp deletion spanning the *psl* operon and 138 additional genes (2.5% of the genome) was detected. This deletion abolishes production of the Psl biofilm component, which may consequently impair biofilm formation. Genes co‐deleted with the *psl* operon contribute to antibiotic resistance: *opdE* disruption enhances imipenem resistance (Huang et al. 1992), while *vqsM* deletion increases resistance to tetracycline/kanamycin (Liang et al. 2014). The absence of *vqsM* and *opdE* genes confers increased antibiotic resistance, providing a clinical adaptive advantage, whereas deletion of the *psl* operon appears incidental and nonadaptive. It was observed that isolate PA326 forms a robust pellicle in microplate wells and does not form adherent biofilm. As the Psl polysaccharide is primarily required for surface attachment, this isolate may be capable of forming a nonadherent biofilm in which the Pel polysaccharide constitutes the main component of the matrix (Zhao et al. 2013).

Another found genetic variant, which, as we supposed, associates with biofilm formation impairment, is the V383G *fleQ* mutation. Information about the effects of *fleQ* mutations is limited (Danecek et al. 2021). The amino acid substitution G240V downregulates flagellar gene expression (Jain and Kazmierczak 2014). Mutations V270G and D302G have been described in *P. aeruginosa* microevolution under microfluidic emulsion conditions (Disney‐McKeethen et al. 2023). The V383G polymorphism is a unique SNP identified in isolate PA108, which exhibited biofilm formation impairment and may therefore underlie the observed phenotype. However, this SNP is not necessarily a loss‐of‐function variant, and additional validation is required to confirm its effect.

The identified frameshift mutations in phosphodiesterases occur in a few percent of genomes in the NCBI genome database. This suggests these mutations may confer a selective advantage for clinical isolates. Deletions of the *psl* operon are also present in NCBI, however, the boundaries of these deletions vary between isolates.

Multiple systems contribute to biofilm formation, with some functional redundancy; thus, the loss of a single component, such as a phosphodiesterase, may not affect biofilm formation, which is consistent with previous findings on *Pseudomonas* species (Dahlstrom et al. 2018; Ha et al. 2014). It seems that only mutations in biofilm matrix synthesis genes or unique regulators lacking functional analogs can lead to defects in biofilm formation in clinical isolates.

However, our conclusions have several limitations. The biofilm‐forming ability of the experimental isolates was assessed using the CV microtiter plate assay under a single experimental condition (37°C, 24 h incubation). Although other assays or growth conditions might yield slightly different results, the chosen parameters are the most commonly used for biofilm formation studies and allow direct comparison with previously published data. The CV assay has a broad specificity for the biofilm matrix and does not account for the number or viability of cells. Nevertheless, different biofilm quantification methods are generally in good agreement with each other (Peeters et al. 2008).

The observed association between the detected mutations and biofilm formation impairment is correlational in nature. Functional verification through gene editing or complementation experiments is required to directly confirm the impact of these mutations. Furthermore, only three isolates were classified as biofilm‐impaired, which limits the statistical power of our analysis. A broader investigation, including additional isolates with biofilm formation impairment, would help clarify the genetic determinants of this phenotype.

## 5 Conclusions

In this study, we identified genetic variants associated with biofilm formation impairment in clinical isolates. These include deletion in the *psl* operon and a nonsense mutation in the *pelA* gene. Additionally, a V383G substitution in FleQ, a transcriptional regulator of biofilm‐related genes, may contribute to biofilm impairment.

Biofilm formation is an important factor contributing to the pathogenicity of *P. aeruginosa*. However, not all clinical isolates are capable of forming biofilms, highlighting the need for further research to understand the adaptive strategies employed by biofilm‐impaired *P. aeruginosa* in the in vivo environment. Identifying the key genetic determinants underlying biofilm impairment may provide valuable insights for the development of antibiofilm therapies targeting essential proteins involved in biofilm formation.

## Author Contributions

**Andrei V. Vvedenskii:** investigation, writing – original draft, writing – review and editing. **Alina S. Ivkina:** methodology. **Dmitry N. Konanov:** investigation, writing – review and editing. **Tatiana A. Savinova:** conceptualization. **Ludmila S. Fedorova:** supervision, writing – review and editing. **Elena N. Ilina:** project administration, supervision, writing – review and editing.

## Ethics Statement

The authors have nothing to report.

## Conflicts of Interest

The authors declare no conflicts of interest.

## Data Availability

Assembled genomes and associated isolation source metadata are accessible via BioProject accession number PRJNA1225146.

## Appendix 1

See Table A1.

**Table A1:** Assembly statistics.

Strain	GenBank WGS master accession	Assembly accession	Source	# Contigs	N50	Length (bp)	GC content	Coverage	# CDS (PGAP)	# Genes (PGAP)	# CDSs (with protein)	# Complete rRNAs (5S, 16S, 23S) (PGAP)	# tRNAs (PGAP)
*Pseudomonas aeruginosa* PA068	JBMAFG000000000	GCA_048567305.1	This study	57	324,817	6,836,575	65.87	148	6335	6402	6238	1, 1, 0	58
*Р. aeruginosa* PA100	JBMAFH000000000	GCA_048567205.1	This study	62	333,728	6,798,481	65.97	151	6296	6361	6200	1, 1, 1	58
*Р. aeruginosa* PA108	JBMAFI000000000	GCA_048567145.1	This study	58	324,817	6,836,879	65.87	130	6337	6404	6240	1, 1, 2	58
*Р. aeruginosa* PA110	JBMAFJ000000000	GCA_048567045.1	This study	78	287,175	7,019,281	65.81	172	6632	6699	6528	1, 1, 1	59
*Р. aeruginosa* PA121	JBMAFK000000000	GCA_048566965.1	This study	68	402,832	6,764,351	66.01	165	6378	6442	6292	1, 1, 1	57
*Р. aeruginosa* PA135	JBMAFL000000000	GCA_048567085.1	This study	80	266,700	6,922,472	66	176	6524	6588	6426	1, 1, 1	57
*Р. aeruginosa* PA144	JBMAFM000000000	GCA_048567185.1	This study	76	331,061	6,923,724	66	144	6522	6586	6,424	1, 1, 1	57
*Р. aeruginosa* PA154	JBMAFN000000000	GCA_048567225.1	This study	99	295,960	7,022,182	65.94	147	6645	6710	6565	1, 1, 1	58
*Р. aeruginosa* PA262	JBMAFO000000000	GCA_048567245.1	This study	54	426,821	6,749,970	66.2	138	6364	6431	6307	1, 1, 1	60
*Р. aeruginosa* PA304	JBMAFP000000000	GCA_048567105.1	This study	70	392,544	6,733,428	65.94	199	6254	6317	6167	1, 1, 1	56
*Р. aeruginosa* PA308	JBMAFQ000000000	GCA_048567025.1	This study	61	508,046	6,477,971	66.33	202	5983	6051	5923	1, 0, 0	58
*Р. aeruginosa* PA312	JBMAFR000000000	GCA_048567125.1	This study	129	229,626	7,168,873	65.95	126	6779	6844	6674	1, 1, 1	58
*Р. aeruginosa* PA313	JBMAFS000000000	GCA_048567165.1	This study	82	285,489	6,922,879	66.02	137	6537	6602	6449	1, 0, 1	57
*Р. aeruginosa* PA315	JBMAFT000000000	GCA_048567005.1	This study	27	674,984	6,413,483	66.47	181	5919	5979	5879	1, 1, 1	53
*Р. aeruginosa* PA321	JBMAFU000000000	GCA_048567345.1	This study	81	307,567	6,919,895	66.02	126	6552	6620	6463	1, 1, 1	61
*Р. aeruginosa* PA326	JBMAFV000000000	GCA_048567065.1	This study	55	520,623	6149390	66.47	127	5694	5756	5660	1, 1, 1	55
*Р. aeruginosa* PA351	JBMAFW000000000	GCA_048567405.1	This study	32	555,642	6,460,106	66.37	153	6032	6094	5986	1, 1, 1	55
*Р. aeruginosa* PA354	JBMAFX000000000	GCA_048567285.1	This study	47	386,157	6,641,410	66.22	180	6214	6278	6157	1, 1, 1	56
*Р. aeruginosa* PA370	JBMAFY000000000	GCA_048567265.1	This study	54	322,440	6,608,031	66.22	155	6209	6271	6138	1, 1, 1	55
*Р. aeruginosa* PA379	JBMAFZ000000000	GCA_048567365.1	This study	60	309,232	6,613,785	66.22	192	6232	6295	6157	1, 1, 1	56
*Р. aeruginosa* PA397	JBMAGA000000000	GCA_048567325.1	This study	44	587,281	6,298,307	66.51	165	5823	5886	5784	1, 1, 1	55

Abbreviations: CDS, coding sequence; GC, guanine‐cytosine; PGAP, prokaryotic genome annotation pipeline; rRNA, ribosomal RNA; tRNA, transfer RNA; WGS, whole‐genome shotgun.
